# Diffusion-controlled on-surface synthesis of graphene nanoribbon heterojunctions†

**DOI:** 10.1039/d2ra01008a

**Published:** 2022-02-25

**Authors:** Christoph Dobner, Gang Li, Mamun Sarker, Alexander Sinitskii, Axel Enders

**Affiliations:** Physikalisches Institut, Universität Bayreuth, Universitätsstraße 30 95447 Bayreuth Germany christoph.dobner@uni-bayreuth.de; Department of Chemistry, University of Nebraska – Lincoln Lincoln NE 68588 USA; Nebraska Center for Materials and Nanoscience, University of Nebraska – Lincoln Lincoln NE 68588 USA

## Abstract

We report a new diffusion-controlled on-surface synthesis approach for graphene nanoribbons (GNR) consisting of two types of precursor molecules, which exploits distinct differences in the surface mobilities of the precursors. This approach is a step towards a more controlled fabrication of complex GNR heterostructures and should be applicable to the on-surface synthesis of a variety of GNR heterojunctions.

## Abstract 2

We report a diffusion-controlled process for the on-surface synthesis of graphene nanoribbon heterojunctions. Differences in the diffusion kinetics of the precursor molecules were exploited to control the GNR architecture.

## Graphical abstract

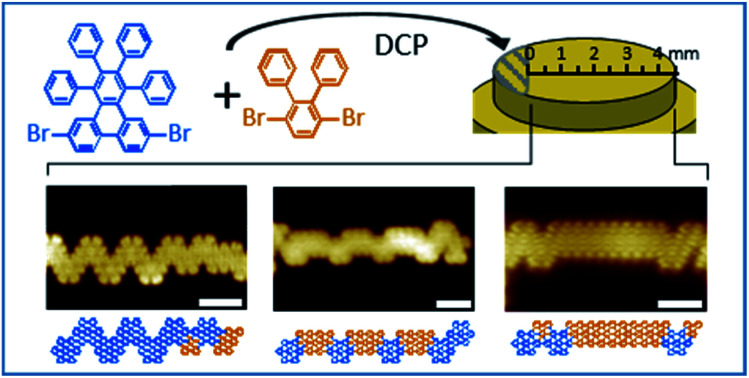

Since the first demonstration of on-surface bottom-up synthesis of graphene nanoribbons (GNR) by Cai *et al.*,^1^ the search for strategies to build functional carbon-based nanostructures with engineered electronic properties has evolved into a very active area of research.^2–4^ Beyond that, more complex GNR-based heterostructures such as semiconductor heterojunctions of both type I and II,^5,6^ junctions with topological interface states,^7^ quantum dots^8^ and p–n-junctions^5^ were reported recently. The design of electronic heterojunctions from GNRs builds on two important principles, which are that (a) the width of the electronic band gap of GNRs can be dependent on their structural width^9–12^ and (b), that the location of the top of the valence band and the bottom of the conduction band in GNRs can be effectively shifted through doping effects.^13–15^

The procedure for on-surface synthesis of GNRs requires the deposition of the precursor molecules on clean, crystalline substrate surfaces under ultrahigh vacuum (UHV), followed by a annealing process to achieve GNR formation by polymerization and cyclodehydrogenation. As it is inherent to any self-assembly strategy, there is not much control over the process itself. The fabrication of heterojunctions for instance, is less a controlled process than it is a mere selection of the desired structure out of random molecular assemblies of all precursor molecules on the surface. While there have been successful attempts to synthesize GNR heterojunctions from precursors with different halogens by taking advantage of their different reactivities,^16^ there is still no universal strategy for a controlled assembly of two types of precursor molecules. The stochastic nature of self-assembly typically yields a broad distribution of hetero ribbons, which are not useful for device fabrication. An important step beyond the randomness in the self-assembly of heterojunction has been achieved by Bronner *et al.* by exploiting the hierarchy of dissociation energies of different halogen groups to built single heterojunction interfaces.^16,17^

In our study we explore an alternative approach. We demonstrate a strategy to gain control over the stoichiometry of hetero GNRs, which are GNRs consisting of two types of precursor molecules, in a single growth experiment. We exploit natural differences in the diffusivity of both types of precursors to create a molecular density gradient with distance from an on-surface reservoir that contains a fixed ratio of both types of precursors. As a direct result, sections of desired length in a single hetero GNR that contain only one type of precursor molecules can be selected as needed at appropriate distances from the reservoir.

We chose for this study the precursor molecules 6,11-dibromo-1,2,3,4-tetraphenyltriphenylene (TPTP), which is commonly used for the synthesis of chevron-type GNR (cGNR),^1,18,19^ as well as 3,6′-dibromo-1,1′′:2′,1′′-terphenyl (DBT), which was used first by Talirz *et al.* to create 9-armchair GNRs (9-AGNR) with straight edges.^4^ cGNR/9-AGNR heterojunctions were very recently subject to extensive theoretical investigation by Taqieddin and Aluru,^20^ where it was predicted that the coupling of chevron- and 9-armchair segments leads to the formation of new, emergent electronic states, neither present in cGNR nor in 9-AGNR, directly at the interfaces due to geometric mismatch. The calculations of 9-armchair segments of varied length, which are sandwiched between chevron segments, predict that the junctions of chevron- and 9-armchair segments are of type I, with a band gap that is dependent on the length as well as on lateral strain. Also, the strongly localized interface states is also susceptible to the 9-armchair segment length. Interface states at GNR heterojunctions such as these and others^7^ are of immense interest, as they represent an opportunity to fine tune their electronic band gap. We show in this work how cGNR/9-AGNR heterojunctions can be fabricated, how surface diffusion of precursor molecules can be exploited to control the architecture of the hetero GNR, and we experimentally confirm a theoretically predicted interface state.

Our concept of diffusion-controlled on-surface synthesis of GNR heterojunctions is based on differences in diffusivity of both precursors, on a single crystal surface. To exploit such differences we deposited a powdered stoichiometric 1 : 1 mix of TPTP and DPT on a 1 mm wide area starting from one sample edge *via* direct contact printing (DCP),^21,22^ as shown in Fig. 1a. This area will serve as a reservoir, or source of molecules, in subsequent diffusion experiments, where we studied molecular aggregates as a function of distance from the deposit using low temperature scanning tunnelling microscopy (STM). The substrate was then annealed after deposition until their known cyclodehydrogenation temperature of 720 K was reached. During annealing, the melting of the deposited molecular crystals was already observed through an optical telescope at temperatures well below the polymerization temperature of 520 K. Activated by the increasing sample temperature the molecules diffuse from this reservoir across the entire surface of the single crystal, and especially over the initially clean surface area where no molecules were deposited.

**Fig. 1:**
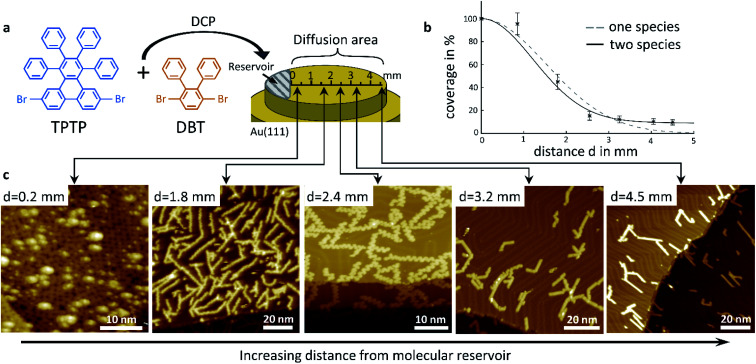
Deposition method and experimental results of density gradient driven GNR self-assembly. (a) Structure of the precursor monomers and schematic principle of deposition. (b) Surface coverage of GNRs plotted against distance d from the location of direct contact. (c) STM images showing different locations along the axis in (a). All images were acquired at *T* = 77 K, *V*_B_ = −2 V, and *I*_T_ = 100 pA.

Representative STM images taken on this area, at distances of 0.2 mm, 1.8 mm, 2.4 mm, 3.2 mm and 4.5 mm away from the edge of the deposited reservoir are shown in Fig. 1c. Fully cyclized GNRs are observable in all images. At close proximity to the reservoir, at a distance of only 0.2 mm from the DCP edge, the STM image shows a densely packed layer of cGNRs, which is covered by a random distribution of 3D objects. At larger distances, all GNRs are within the first layer and in direct contact with the substrate, and their areal density is decreasing with increasing distance from the reservoir. Even at a large distance of 4.5 mm away from the edge of the DCP area we observe a surface coverage of approximately 10% with GNRs.

Closer inspection of the STM images shows that the relative concentrations of cGNR segments and 9-AGNR segments change with distance from the DCP area too. The GNRs in the first layer at a distance of 0.2 mm are exclusively cGNRs, whereas at the largest distance of 4.5 mm we only find 9-AGNRs. At intermediate distances, the ribbons are typically hetero GNRs consisting of cGNR segments as well as of 9-AGNR segments. The cGNR fraction in those ribbons decreases with increasing distance from the DCP area.

We conclude, based on the presented STM images, that the diffusivity of the 9-AGNR-forming monomers of DPT is considerably higher than the diffusivity of the cGNR-forming TPTP monomers, such that 9-AGNRs can be found at distances that are out of reach for TPTP within our experimental timescale. It is striking from observation of Fig. 1c, that very near the DCP area we observe exclusively cGNRs with unreacted 3D-adsorbates, most likely DPT clusters, sitting on top of them. It thus appears that besides differences in the diffusivity there are also differences in the polymerization temperatures and in the catalytic benefits for both types of molecules by the substrate.

Before analyzing the hetero GNR architecture in detail, we discuss the specifics of molecular diffusion, as this is key to the diffusion-controlled on-surface synthesis of GNR heterojunctions. We determined the total surface coverage, which includes both precursor species, from a graphical analysis of our STM images such as those in Fig. 1c and others. The surface coverage as function of distance from the DCP area is summarized in Fig. 1b. The graph reflects clearly the visual impression that the overall surface coverage of GNRs decreases with increasing distance. The errors in coverage are the result of poor statistics and of reduced spatial resolution in some of the images. At sufficiently low surface coverage with molecules their diffusion can be described by a random walk using the Einstein–Smoluchowsky equation. However, this model loses its validity when the movement of the molecules is no longer independent. This is the case at high surface coverage when the diffusion of molecules is kinetically hindered. As was derived by Fick in 1855,^23^ it is the local density gradient that acts as a driving force on molecular motion, which in our case is the molecule reservoir at the DCP area. The resulting coverage as function of distance was modelled using Fick's second law, and the resulting fit to our data is shown in Fig. 1b, along with the experimental data, as a dashed line, assuming that there is only one diffusion coefficient that applies to all molecules on the surface. Notably this simple model does not reproduce our experimental data well, especially at larger distances, where the coverage decreases more slowly than our model would predict. A much better fit to our data at larger distance, shown as continuous line, can be achieved by assuming a double exponential decay based on two different diffusion constants, one for each species in the 1 : 1 molar mixture of precursor molecules. It should be noted that this model is very simple and does not account for any kind of interaction with surface defects or other aggregates. Yet, it is consistent with our conclusion that DPT precursors have a considerably higher surface diffusivity than TPTP precursors.

Hetero GNRs, which include one or more cGNR/9-AGNR heterojunctions, can predominantly be found in a region on the substrate surface at distances between 2–4 mm from the DCP area. High-resolution STM images shown representative hetero GNRs, along with a structure model, are shown in Fig. 2. The fraction of 9-AGNR increases in the images from a to d, as these images were collected at distances between 2 and 4 mm from the DCP area. Fig. 2b–d show hetero ribbons containing segments of cGNR and 9-AGNR. The 9-AGNR segment is particularly long in d, and the cGNR segments in c are particularly short as they consist of only one TPTP monomer. Interestingly, Fig. 2a shows a new, unexpected heterojunction of a cGNR and another chevron-type GNR segment of smaller width. This narrower chevron-type GNR appears to be new and is formed by the alternating arrangement of TPTP and DTP monomers, as is shown in the corresponding structure model. Compared to a cGNR, which is typically 2.3 nm wide (15 carbon atoms) the narrow cGNR is only 1.9 nm wide (12 carbon atoms). By comparison, the 9 carbon atoms wide 9-AGNR segments are 1.5 nm wide.

**Fig. 2:**
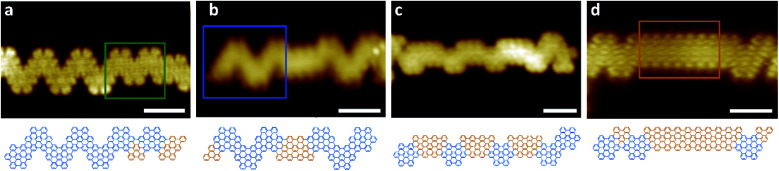
STM images and respective structure models of select GNR heterojunctions. Marked with colored rectangles are pristine cGNR (blue), 9-AGNR (orange) and hybrid structures (green). All images were acquired at *T* = 77 K (a: *V*_B_ = −2 V, *I*_T_ = 1 nA; b: *V*_B_ = −1.65 V, *I*_T_ = 500 pA; c: *V*_B_ = −2 V, *I*_T_ = 1 nA; d: *V*_B_ = −1.4 V, *I*_T_ = 500 pA). Scale bars are all 2 nm.

Electronic properties of a select cGNR/9-AGNR junction, shown in Fig. 3a, were examined using scanning tunnelling spectroscopy. The local point spectra Fig. 3b, taken on 9-AGNR and on cGNR segments, agree well with spectroscopic data published in the literature. Specifically, they reflect the known electronic band gap of 1.4–1.5 eV for 9-AGNR^11,24^ as well as 2.4–2.5 eV for cGNR.^14,17^ The straddling band alignment at the interface of cGNR and 9-AGNR segments corresponds to that of a type I junction. To investigate local electronic states at the interface of cGNR and 9-AGNR segments we recorded local density of states (LDOS) maps of the heterojunction, see Fig. 3c. The maps show distinctly the spatial distribution of the LUMO of the 9-AGNR segment at +1.45 eV, which is located along the 9-AGNR edges, as well as the spatial distribution of the HOMO of the cGNR, located along the cGNR edges, both in agreement with theoretical predictions^20^ and published experimental data.^16^ New here is the observation of strong electronic localization at the concave kink of the chevron segment immediately at the interface to the 9-armchair segment, which is at approximately −0.65 eV energetically near the HOMO of the cGNR (−0.7 eV^16,24^). This electronic localization in the HOMO map is attributed to a computationally predicted interfacial state at the interface of the chevron- and 9-armchair segment.^20^

**Fig. 3:**
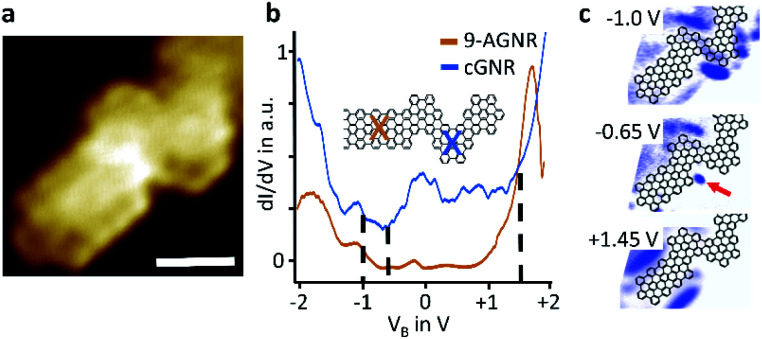
Measurements and computational results of electronic properties of a selected heterojunction. (a) STM image of the select heterojunction (*T* = 77 K, *V*_B_ = −0.1 V, *I*_T_ = 500 pA, scalebar = 2 nm) (b) d*I*/d*V* point spectra acquired at the marked locations on the GNR. Data were normalized and offset slightly for clarity. (c) d*I*/d*V* maps at different voltages where blue represents high intensity (*T* = 77 K, *V*_mod_ = 50 mV, *f*_mod_ = 865 Hz, *I*_T_ = 500 pA). The red arrow highlights the interfacial state.

In summary, we showed that TPTP and DPT molecules form hetero GNRs on the (111) surface of Au, which contain segments of both types of molecules, *i.e.* chevron-type segments and 9-armchair segments. However, both types of molecules have vastly different diffusivities on the Au(111) surface at moderate annealing temperatures, with 9-armchair-forming DPT moving over significantly larger distances within a given annealing time than TPTP. Such molecule-specific diffusion constants form the basis of a new on-surface diffusion-controlled synthesis approach for hetero GNRs with controlled stoichiometry. Since there is a wealth of examples in current research where complex self-assembled nanostructures were formed on surfaces by exploiting adsorbate diffusivity on flat surfaces as well as on nanotemplated surfaces, we speculate here that also the self-assembly of GNRs and GNR heterostructures could be, at least to some degree, be further controlled *via* template surfaces. A significant advantage of the presented diffusion-controlled hetero GNR synthesis is that a large number of hetero GNRs with a stoichiometry gradient across the substrate surface is produced within a single shot experiment, allowing for big-data type research on surface supported GNR heterostructures. A theoretically predicted interface state at the interface of 9-armchair and chevron segments, near the HOMO of the chevron segment, was experimentally confirmed. The confirmation of this interface state is significant, as emergent interface states such as this can help control the overall band gap of the heterojunction.

## Conflicts of interest

There are no conflicts of interest to declare.

## Supplementary Material

RA-012-D2RA01008A-s001
