# Supplementary material for: Diffusion-controlled on-surface synthesis of graphene nanoribbon heterojunctions
Source: RSC Adv. 2022 Feb 25;12(11):6615–8. doi: 10.1039/d2ra01008a (PMC8981978; doi:10.1039/d2ra01008a)
Supplement: RA-012-D2RA01008A-s001 [file RA-012-D2RA01008A-s001.pdf]

Electronic Supplementary Material (ESI) for RSC Advances.
This journal is © The Royal Society of Chemistry 2022

Electronic Supplementary Information (ESI) for

Diffusion-controlled on-surface synthesis of graphene nanoribbon heterojunctions

Christoph Dobner,[a,*] Gang Li,[b] Mamun Sarker,[b] Alexander Sinitskii,[bc] and Axel Enders[a]

a. Physikalisches Institut, Universität Bayreuth, Universitätsstraße 30, 95447 Bayreuth, Germany
b. Department of Chemistry, University of Nebraska – Lincoln, Lincoln, NE 68588, USA
c. Nebraska Center of Materials and Nanoscience, University of Nebraska – Lincoln, Lincoln, NE 68588, USA

*E-mail: christoph.dobner@uni-bayreuth.de

Methods

DCP Preparation.

A 1:1 molar mixture of both precursor was prepared under ambient conditions using a Mettler AE160 high precision balance and subsequently sonicated for 10 minutes to ensure a homogeneous mixture. The stamp, consisting of a piece of cotton fibre, cleaned by placing it in an ethanol bath and sonicating it for several minutes, attached to a standard sample plate, was then dipped in the molecular mixture and inserted in the LoadLock of the UHV system for degassing.

On-surface Synthesis and Analysis.

All experiments were performed in ultrahigh vacuum (UHV) conditions. The Au(111) substrate used for the on-surface synthesis was cleaned prior to deposition by several standard sputtering-annealing cycles. A 1:1 molar mixture of both molecular precursors was deposited via direct contact printing (DCP) onto one side of the crystal surface. Subsequent post annealing completed the synthesis process. For analysis of the sample we used a low-temperature scanning tunnelling microscope (Scienta Omicron) attached directly to the preparation chamber. All measurements were performed under a base pressure of $< 10^{-10}$ mbar and at 77 K. The spectroscopy data was obtained using a lock-in amplifier setup with a modulation frequency of 865 Hz and an amplitude of 50 mV to modulate the sample bias and measure dI/dV directly.
